# Discovery-Based Science Education: Functional Genomic Dissection in Drosophila by Undergraduate Researchers

**DOI:** 10.1371/journal.pbio.0030059

**Published:** 2005-02-15

**Authors:** Jiong Chen, Gerald B Call, Elsa Beyer, Chris Bui, Albert Cespedes, Amy Chan, Jenny Chan, Stacy Chan, Akanksha Chhabra, Peter Dang, Artemis Deravanesian, Brenda Hermogeno, James Jen, Eunha Kim, Eric Lee, Gemma Lewis, Jamie Marshall, Kirsten Regalia, Farnaz Shadpour, Aram Shemmassian, Kristin Spivey, Maggie Wells, Joy Wu, Yuki Yamauchi, Amir Yavari, Anna Abrams, Amanda Abramson, Latiffe Amado, Jenny Anderson, Keenan Bashour, Elena Bibikova, Allen Bookatz, Sarah Brewer, Natalie Buu, Stephanie Calvillo, Joseph Cao, Aileen Chang, Daniel Chang, Yuli Chang, Yibing Chen, Joo Choi, Jeyling Chou, Sumit Datta, Ardy Davarifar, Poonam Desai, Jordan Fabrikant, Shahbaz Farnad, Katherine Fu, Eddie Garcia, Nick Garrone, Srpouhi Gasparyan, Phyllis Gayda, Chad Goffstein, Courtney Gonzalez, Mariam Guirguis, Ryan Hassid, Aria Hong, Julie Hong, Lindsay Hovestreydt, Charles Hu, Farid Jamshidian, Katrin Kahen, Linda Kao, Melissa Kelley, Thomas Kho, Sarah Kim, Yein Kim, Brian Kirkpatrick, Emil Kohan, Robert Kwak, Adam Langenbacher, Santino Laxamana, Chris Lee, Janet Lee, So-Youn Lee, To Hang S Lee, Toni Lee, Sheila Lezcano, Henry Lin, Peter Lin, Julie Luu, Thanh Luu, Will Marrs, Erin Marsh, Sarah Min, Tanya Minasian, Amit Misra, Miles Morimoto, Yasaman Moshfegh, Jessica Murray, Cynthia Nguyen, Kha Nguyen, Ernesto Nodado, Amanda O'Donahue, Ndidi Onugha, Nneka Orjiakor, Bhavin Padhiar, Mara Pavel-Dinu, Alex Pavlenko, Edwin Paz, Sarah Phaklides, Lephong Pham, Preethi Poulose, Russell Powell, Aya Pusic, Divi Ramola, Meghann Ribbens, Bassel Rifai, Desiree Rosselli, Manyak Saakyan, Pamela Saarikoski, Miriam Segura, Ramnik Singh, Vivek Singh, Emily Skinner, Daniel Solomin, Kosha Soneji, Erika Stageberg, Marina Stavchanskiy, Leena Tekchandani, Leo Thai, Jayantha Thiyanaratnam, Maurine Tong, Aneet Toor, Steve Tovar, Kelly Trangsrud, Wah-Yung Tsang, Marc Uemura, Mary Unkovic, Emily Vollmer, Emily Weiss, Damien Wood, Sophia Wu, Winston Wu, Qing Xu, Kevin Yackle, Will Yarosh, Laura Yee, George Yen, Grant Alkin, Sheryllene Go, Devon M Huff, Helena Minye, Eric Paul, Nikki Villarasa, Allison Milchanowski, Utpal Banerjee

## Abstract

How can you combine professional-quality research with discovery-based undergraduate education? The UCLA Undergraduate Consortium for Functional Genomics provides the answer

The excitement of scientific research and discovery cannot be fully conveyed by didactic lectures alone. Several recent initiatives and proposals, therefore, have supported a more participatory, discovery-based instruction for undergraduate science education [1,2]. In functional genomics, we have found an ideal platform to simultaneously benefit students and contribute to scientific discovery. The sequencing of eukaryotic genomes has facilitated the identification of complete sets of genes in humans and model genetic organisms. This has allowed many forms of high-throughput analyses of transcriptional profiles, protein interactions, structural motifs, and even genome-wide knock-downs in cell lines or in selected organisms. However, one of the best tools to provide functional information about gene action— obtaining in vivo evidence about the phenotype resulting from heritable loss of function—is difficult and less amenable to high-throughput research. We were able to achieve a large-scale in vivo analysis with a significant number of undergraduate students at UCLA, called the UCLA Undergraduate Consortium for Functional Genomics. This work, a practical manifestation of policy positions proposing discoverybased education, is described in summary form here (and in Box 1) and in detail online at http://www.bruinfly.ucla.edu. This effort combines professional-quality research with a strategy for research-based undergraduate education.

**Box 1. Scientific Results**

The Drosophila eye is an intricate neurocrystalline lattice of approximately 800 individual ommatidia arrayed in a very precise order (Figure 3A) [3]. Minor perturbations in ommatidial development can be easily detected, making it a very sensitive system for functional genomic screens [3]. Our study utilized 1,375 unique recessive lethal transposable element (P-element) insertion stocks from the 2nd and 3rd chromosomes of Drosophila to characterize their later role in eye development. To avoid early lethality, the FLP/FRT system was used to generate homozygous mutant tissue specifically in the eye [4]. Of the mutations analyzed, 501 (36%) displayed a mutant eye phenotype, providing the first genome-wide estimate of the fraction of essential genes that are also involved in eye development. Adult eye phenotypes were classified into three broad classes: rough, cell lethal, and glossy (Figure 3). The genes responsible for these phenotypes were assigned into 19 different functional categories, which are summarized in Table 1. Signal transduction components previously established to be important for eye development (e.g,. *EGFR*, *pointed*, *Star*, *tramtrak*, *Delta*) were identified, validating the effectiveness of our screen. In addition, our genomics approach has shown that a number of novel classes of genes are involved in eye development that have not been previously described (Table 1).

**Figure 3:**
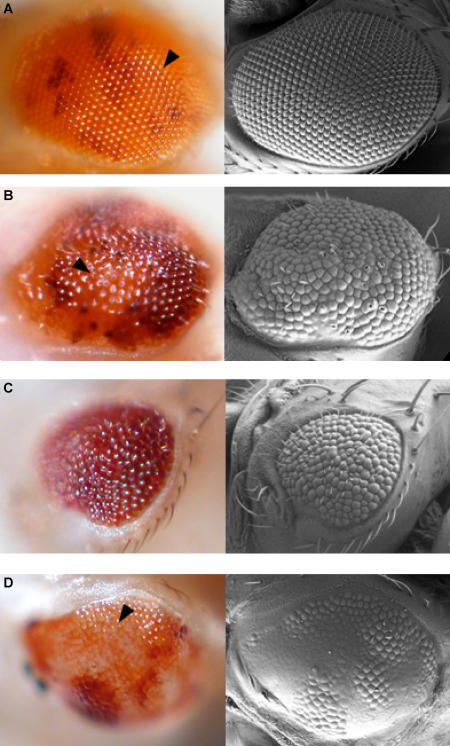
Summary of Phenotypes Determined Light (left panels) and scanning electron (right panels) micrographs of mosaic Drosophila eyes. Large homozygous mutant clones are orange (arrowheads); heterozygous tissues are dark red. Examples are shown of lethal mutations that give a (A) wild-type (63.4% of lethal mutations), (B) rough (disordered ommatidia, 18.2%), (C) cell lethal (absence of homozygous mutant tissue, 14.5%), and (D) glossy (loss of lens structure, 3.9%) phenotype. For details on how clones are generated, see http://www.bruinfly.ucla.edu.

**Table 1:**
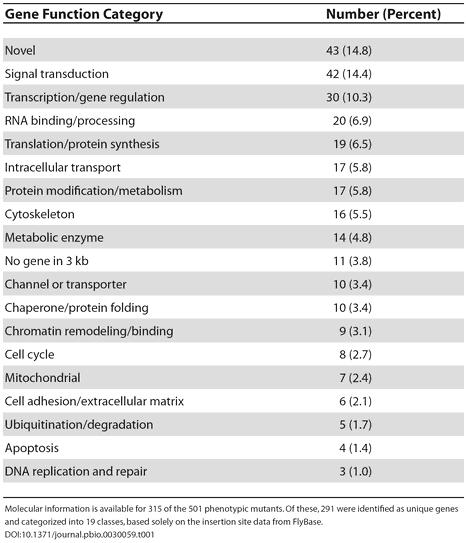
Summary of Genes Involved in Adult Eye Development

Molecular information is available for 315 of the 501 phenotypic mutants. Of these, 291 were identified as unique genes and categorized into 19 classes, based solely on the insertion site data from FlyBase

We have created a novel curriculum with three main components: didactic, computer, and laboratory. The only prerequisite for this course is high school advanced-placement-level biology; all other knowledge necessary for the course is taught within it. Since there are no other prerequisites for the course, a majority of the students enrolled are freshmen and sophomores, enabling us to educate them in this novel way early in their undergraduate career. Approximately 30 students take this course each quarter, and it is offered every quarter through the school year, allowing for a broad impact. In the lecture series, students are exposed to interactive lectures on background material, basic concepts of genetics, research ethics, and career options. For their “midterm,” each student proposes an experiment in a grant proposal formatted according the National Institutes of Health requirements. The “final” is written as a scientific paper summarizing the student's own results. In the computer section, students perform research with a “virtual fly lab” to help them understand more about their crosses in the laboratory section. In addition, they learn about modern genomic resources available on the Internet, and utilize some of the genomics tools available (e.g., BLAST) to help them determine the identity and function of their disrupted genes. The main component of the class, however, is the laboratory portion.

In the laboratory, the students perform all the necessary work to manipulate the genotypes of their stocks to determine what effect homozygous mutation of their target genes has in the adult Drosophila eye (Figure 1). To accomplish this, the students perform five-generation Drosophila crosses that nicely fit into a ten-week quarter. Each student is assigned about ten mutants to work with. During the quarter, students are able to recombine each mutation onto a flippase (FLP) recombination target (FRT) chromosome, generate mutant somatic clones, and record details of the adult eye phenotype with both light and scanning electron microscopic techniques. The students then upload their data into an online database (http://www.bruinfly.ucla.edu).

**Figure 1:**
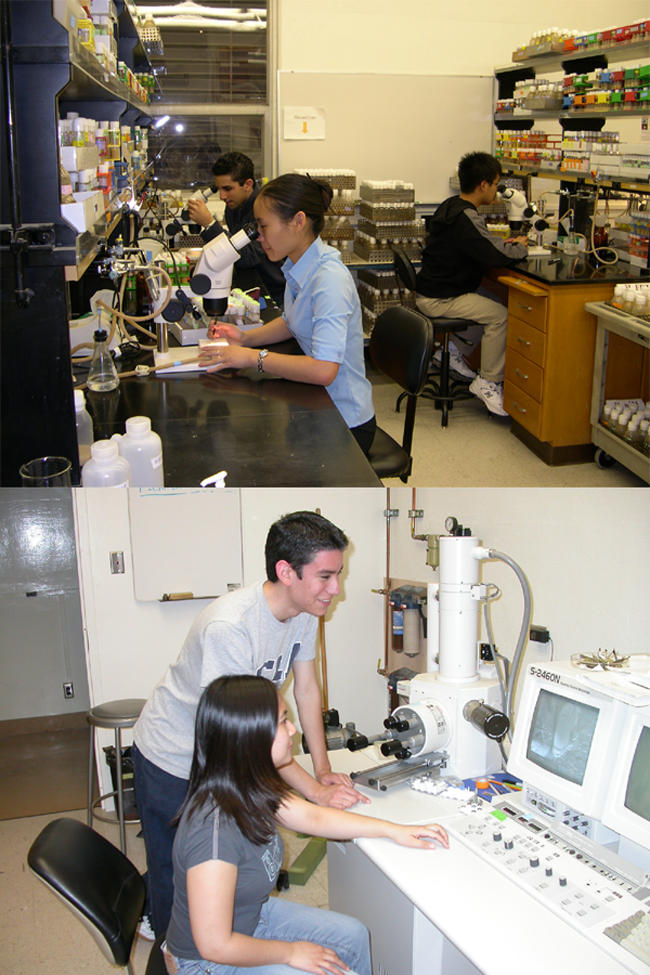
Representative Pictures from the Laboratory Section of the Course

Our database contains pictures of the mutant eyes for all of the stocks examined, as well as other information pertinent to that stock, including the gene disrupted, the exact genomic location of the P-element insertion, and whether an excision of the P-element has been performed and its results. A sample Web page of the database is shown in Figure 2. Following the introductory course, a small number of students continue to analyze the developmental basis for select mutations in future quarters in more advanced laboratory classes. In these advanced classes, the students perform P-element excision experiments to determine whether the mutant phenotype observed is indeed derived from the P-element. These students have performed 294 excision experiments, the results of which indicate that 72% of the stocks successfully revert to wildtype phenotype when the P-element is removed. Over the last two years, we have educated 138 students in the introductory course. Advanced classes have totaled 96 student-quarters (46 students, each working two or more additional quarters).

**Figure 2:**
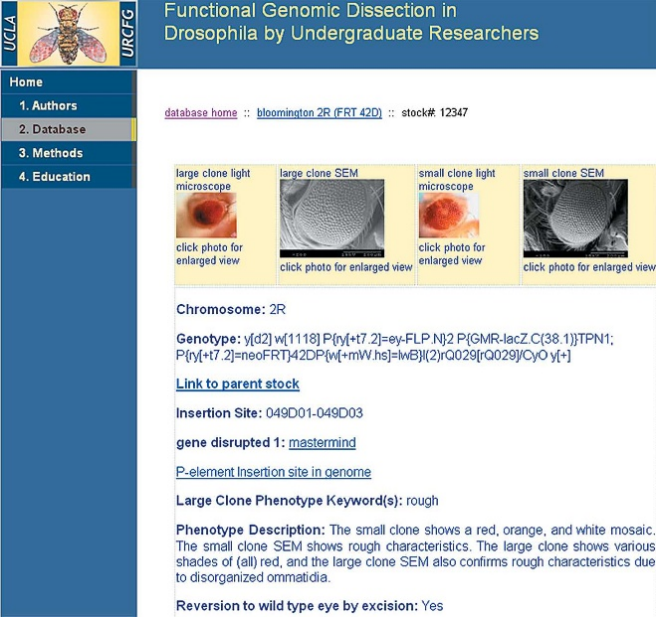
Example of the Type of Data Available from the Online Database (http://www.bruinfly.ucla.edu)

In summary, discovery-based experiments in functional genomics are well suited for undergraduate education: they actively engage a large number of students in research without compromising their didactic training. The sense of ownership developed from this research amplifies the students' learning experience. For the research community, the online database and the large collection of newly generated FRT-lethal lines represent a valuable resource for future experiments in eye development. Furthermore, the stocks developed can be used to create mutant clones in an investigator's tissue of choice. This novel approach for performing research, for which functional genomics is very amenable, not only encourages many students in new ways of thinking but also generates professional-quality results and resources for the scientific community.

## Supporting Information

Figure S1 A Compilation of the Undergraduates and Some of Their Fly Mutants (1.2 MB JPG).
