# Supplementary figures and images for: Discovery-Based Science Education: Functional Genomic Dissection in Drosophila by Undergraduate Researchers
Source: PLoS Biol. 2005 Feb 15;3(2):e59. doi: 10.1371/journal.pbio.0030059 (PMC548953; doi:10.1371/journal.pbio.0030059)

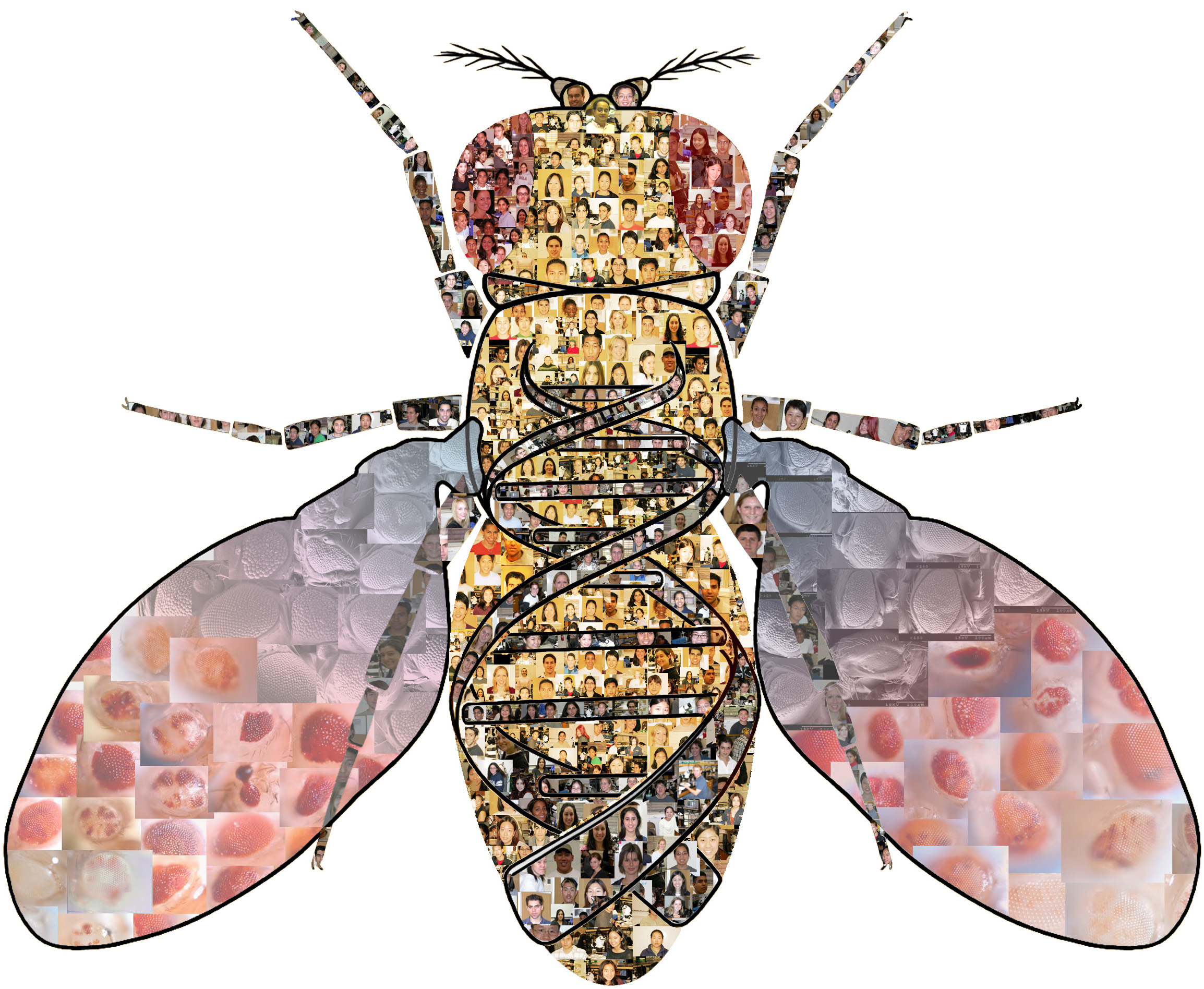

Supplement: Figure S1 — (1.2 MB JPG). [file pbio.0030059.sg001.jpg]
